# Dynamics of Rubisco regulation by sugar phosphate derivatives and their phosphatases

**DOI:** 10.1093/jxb/erac386

**Published:** 2022-09-29

**Authors:** Douglas J Orr, Alice K J Robijns, Christopher R Baker, Krishna K Niyogi, Elizabete Carmo-Silva

**Affiliations:** Lancaster Environment Centre, Lancaster University, Lancaster LA1 4YQ, UK; Lancaster Environment Centre, Lancaster University, Lancaster LA1 4YQ, UK; Howard Hughes Medical Institute, Department of Plant and Microbial Biology, University of California, Berkeley, CA, USA; Howard Hughes Medical Institute, Department of Plant and Microbial Biology, University of California, Berkeley, CA, USA; Molecular Biophysics and Integrated Bioimaging Division, Lawrence Berkeley National Laboratory, Berkeley, CA, USA; Lancaster Environment Centre, Lancaster University, Lancaster LA1 4YQ, UK; University of Essex, UK

**Keywords:** CA1P, CA1Pase, dynamic regulation, Rubisco, Rubisco activase, sugar phosphates, XuBP, XuBPase

## Abstract

Regulating the central CO_2_-fixing enzyme Rubisco is as complex as its ancient reaction mechanism and involves interaction with a series of cofactors and auxiliary proteins that activate catalytic sites and maintain activity. A key component among the regulatory mechanisms is the binding of sugar phosphate derivatives that inhibit activity. Removal of inhibitors via the action of Rubisco activase is required to restore catalytic competency. In addition, specific phosphatases dephosphorylate newly released inhibitors, rendering them incapable of binding to Rubisco catalytic sites. The best studied inhibitor is 2-carboxy-d-arabinitol 1-phosphate (CA1P), a naturally occurring nocturnal inhibitor that accumulates in most species during darkness and low light, progressively binding to Rubisco. As light increases, Rubisco activase removes CA1P from Rubisco, and the specific phosphatase CA1Pase dephosphorylates CA1P to CA, which cannot bind Rubisco. Misfire products of Rubisco’s complex reaction chemistry can also act as inhibitors. One example is xylulose-1,5-bisphosphate (XuBP), which is dephosphorylated by XuBPase. Here we revisit key findings related to sugar phosphate derivatives and their specific phosphatases, highlighting outstanding questions and how further consideration of these inhibitors and their role is important for better understanding the regulation of carbon assimilation.

## Teaser

We review the complex regulation of Rubisco by sugar phosphate derivatives and their phosphatases, and highlight unresolved questions for a better understanding of the regulation of carbon assimilation.

## Introduction

Rubisco activity is regulated by multiple factors in the chloroplast, including changes in the capacity to regenerate the substrate ribulose-1,5-bisphosphate (RuBP), the availability of CO_2_ and Mg^2+^ which affects the carbamylation status, the presence and activity of ancillary proteins, and inhibitory compounds that bind Rubisco catalytic sites preventing activity (Bracher *et al.*, 2017). To be catalytically competent, catalytic sites need to form a stable carbamate by sequential binding of ‘activator’ CO_2_ and Mg^2+^, prior to binding the sugar phosphate substrate RuBP. Initiation of either a carboxylation or an oxygenation reaction then commences via an attack on the substrate by CO_2_ or O_2_, respectively (Bracher *et al.*, 2017). Once carbamylated, the catalytic site can become inhibited by the binding of several compounds similar in structure to RuBP. Similarly, if RuBP binds to the catalytic site before carbamylation, it can effectively act as an inhibitor (Carmo-Silva *et al.*, 2015), because catalysis cannot take place and the catalytic site adopts a closed, unproductive conformation. Inhibition of Rubisco catalytic sites is modulated by environmental cues; for example, the binding of RuBP to uncarbamylated sites plays a significant inhibitory role at low light (Perchorowicz *et al.*, 1981), and the production of inhibitory misfire products of Rubisco catalysis increases with temperature (Kim and Portis, 2004; Salvucci and Crafts-Brandner, 2004; Schrader *et al.*, 2006). The extent to which each inhibitor limits Rubisco activity depends on the species and the chloroplast stromal environment, including the concentrations of CO_2_, Mg^2+^, and the various sugar phosphates.

Rubisco activase (Rca) uses energy from ATP hydrolysis to reconfigure Rubisco catalytic sites and facilitate the release of inhibitors (see reviews by Carmo-Silva *et al.*, 2015; Bracher *et al.*, 2017; Mueller-Cajar, 2017; Shivhare and Mueller-Cajar, 2018). Once released from catalytic sites, dephosphorylation of sugar phosphate derivatives by a phosphatase prevents these from binding another catalytic site, and catalytic site carbamylation ensures productive binding of RuBP. Rubisco activity can therefore be modulated by reversible carbamylation and/or by tight binding and release of sugar phosphate derivatives from catalytic sites. The degree to which each mechanism is employed depends on the species, with most plants employing a combination of both (Sage *et al.*, 1993). Most inhibitors of Rubisco are sugar phosphate derivatives, ranging from compounds that are actively synthesized through to Rubisco reaction misfire products (summarized in Table 1).

**Table 1.** Summary of key sugar phosphate inhibitors of Rubisco activity, with comparison with the substrate RuBP.

**Name**	**Structure**	**Source**	**Role**	**Phosphatase**
2-Carboxy-d-arabinitol 1-phosphate (CA1P)	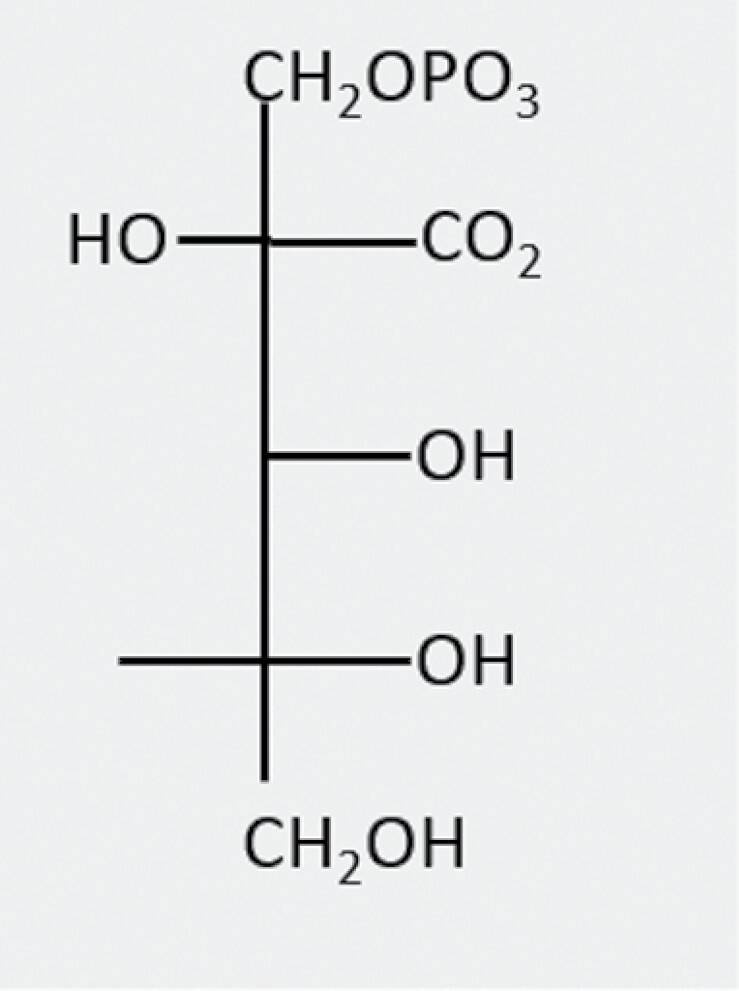	Produced in low light/darkness from CA	Light/dark regulation of Rubisco activity	CA1Pase
Xylulose 1,5-bisphosphate (XuBP)	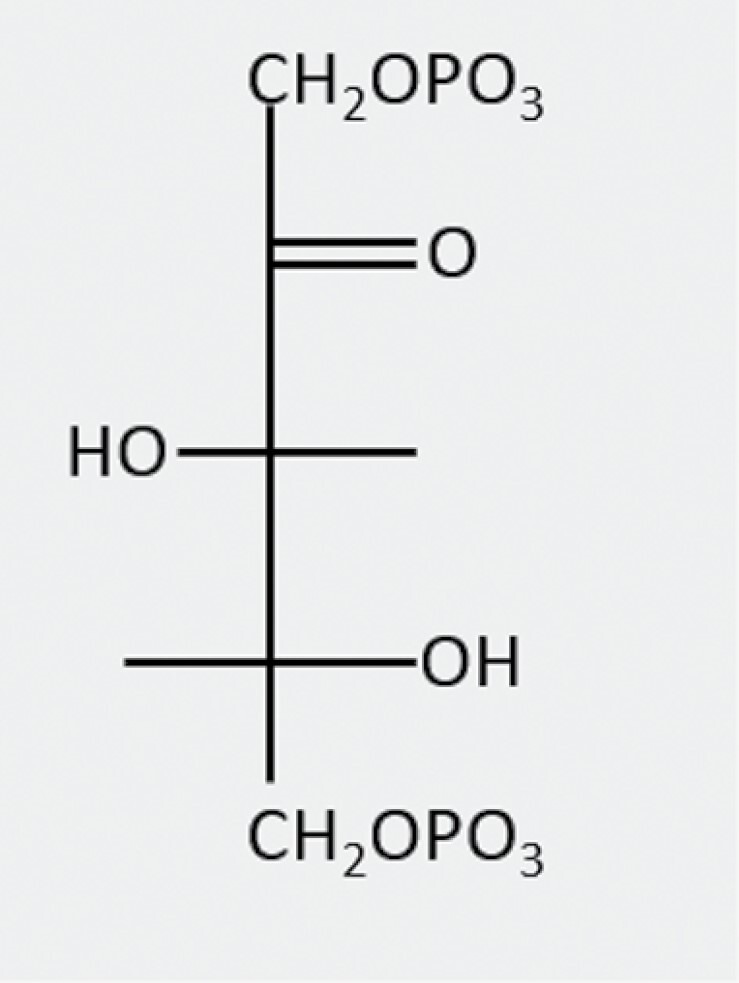	Misfire product of Rubisco carboxylation	?	XuBPase
d-Glycero-2,3-pentodiulose 1,5-bisphosphate (PDBP)	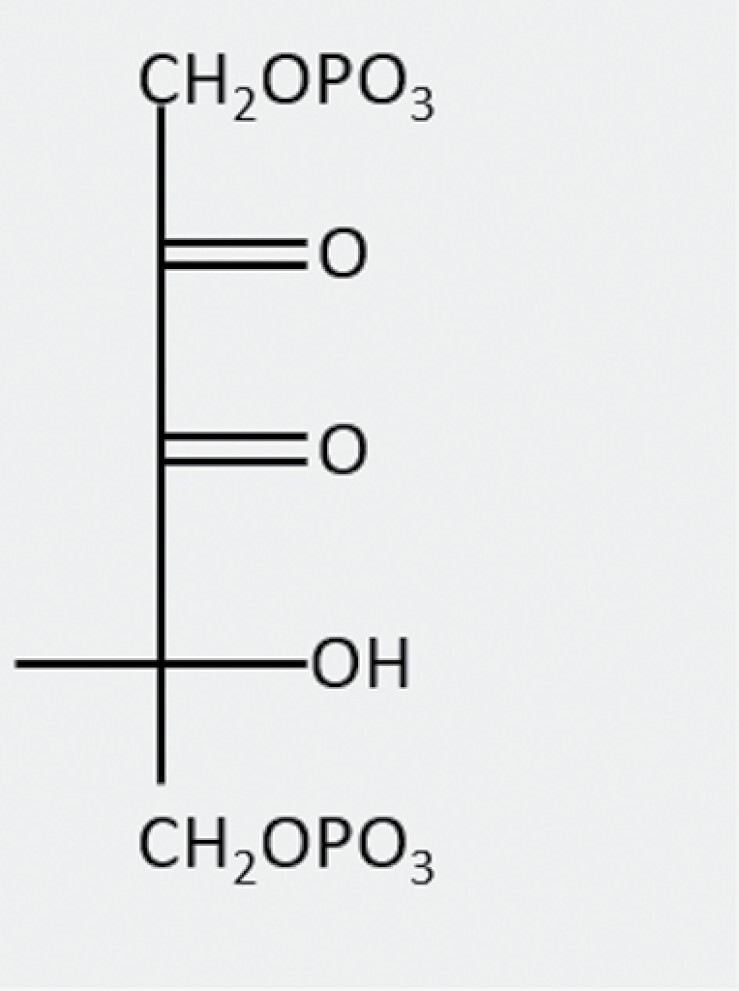	Misfire product of Rubisco oxygenation	?	Can be dephosphorylated by CA1Pase
Carboxy-tetritol-1,5-bisphosphate (CTBP)	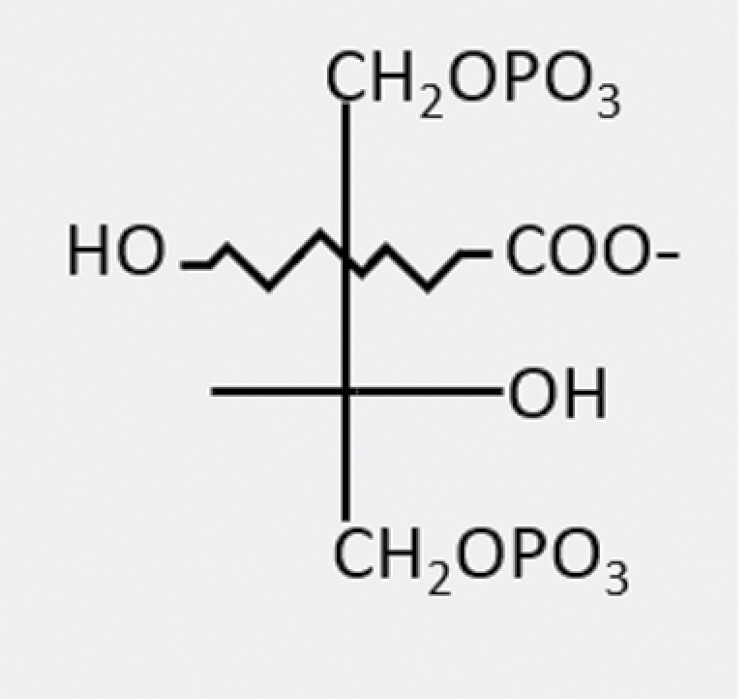	Rearrangement of PDBP	?	?
Ribulose-1,5-bisphosphate (RuBP)	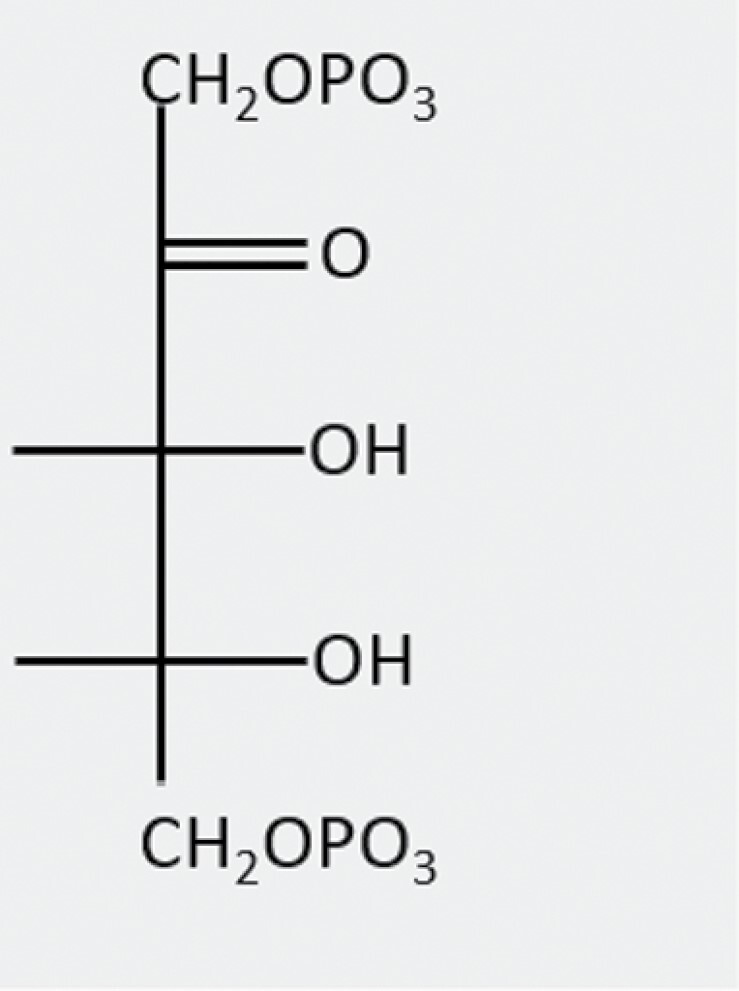	Calvin–Benson–Bassham cycle	Substrate (inhibits non-carbamylated catalytic sites)	n/a

The mechanism and physiological significance of Rubisco regulation by inhibitors remain poorly understood, limiting assessment of whether it may be a target for improved crop productivity and sustainability in the agricultural context (Parry *et al.*, 2008; Andralojc *et al.*, 2012). The study of Rubisco inhibitors has been hampered by their highly similar chemical structures, along with difficulties in accurately determining the low abundance of certain Rubisco misfire products (Keys *et al.*,1995; Andralojc *et al.*, 2002; Pearce, 2006). Historically, research has focused on 2-carboxy-d-arabinitol 1-phosphate (CA1P) and how it dynamically regulates Rubisco activity in concert with changes in light conditions. This regulation is associated with longer periods of shade (>30 min), whereas short shade periods are more likely associated with rapid carbamylation/decarbamylation of Rubisco (Taylor *et al*., 2022). CA1P-induced dark inhibition of Rubisco is currently thought to be present in all C_3_ plants to some degree, with mixed observations in other photosynthetic subtypes. Despite being first mentioned nearly three decades ago (Portis, 1995), only more recently has work begun to decipher the role of xylulose-1,5-bisphosphatase (XuBPase), responsible for rendering the misfire product xylulose-1,5-bisphosphate (XuBP) non-inhibitory (Bracher *et al.*, 2015). In this review, we revisit key findings relating to sugar phosphate derivatives that inhibit Rubisco activity and to their phosphatases, highlight outstanding questions, and hypothesize how further consideration of these inhibitors and their role could be important for better understanding the regulation of Rubisco and to maximize the efficiency of carbon assimilation.

## Synthesis and abundance of CA1P, a nocturnal inhibitor of Rubisco activity

**Fig. 1.**
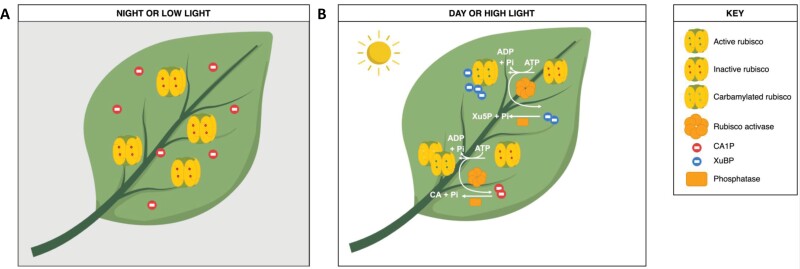
Dynamics of Rubisco inhibition during low and high light. (A) At night, CA1P accumulates in the chloroplast. CA1P inhibits Rubisco activity by binding tightly to Rubisco catalytic sites. (B) In the light, inhibitors such as CA1P or misfire products such as XuBP are removed by Rubisco activase. These sugar phosphates are then dephosphorylated by specific phosphatases that render them non-inhibitory.

The tight binding of CA1P to Rubisco during low light or darkness, and its removal during high light, generates a characteristic diurnal pattern of Rubisco activity, whereby the enzyme is inhibited at night or at low light, and active during the day or at high light (Fig. 1). CA1P-bound Rubisco catalytic sites are reactivated by two light-activated stromal enzymes: first, Rca removes the CA1P molecule, freeing the catalytic site for catalysis (Robinson and Portis, 1988; Heo and Holbrook, 1991). Then, 2-carboxy-d-arabinitol 1-phosphatase (CA1Pase) removes the phosphate group of CA1P, resulting in non-inhibitory 2-carboxy-d-arabinitol (CA) (Holbrook *et al.*, 1989; Moore *et al.*, 1991; Moore and Seemann, 1992).

CA1P is the only known Rubisco inhibitor that is actively synthesized (Gutteridge *et al.*, 1986), via the phosphorylation of CA. CA synthesis is itself linked to the Calvin–Benson–Bassham cycle, with strong evidence that it derives from the intermediate FBP (fructose-1,6-bisphosphate). The observed structural similarity between hamamelose-2,5-bisphosphate (HBP) and CA1P (Beck *et al.*, 1989) agrees with the demonstration through ^14^C labelling of the potential for FBP to be converted to HBP, and that dephosphorylation of HBP could then produce hamamelose (Gilck *et al.*, 1974). Subsequent experimental evidence for the conversion of ^14^C-labelled hamamelose exclusively into CA in the light, and both CA and CA1P in the dark (Moore and Seemann, 1990; Moore *et al.*, 1991; Andralojc *et al.*, 2002), provides strong evidence for the proposed pathway, which is further validated by work with antisense FBPase potato plants that accumulated higher levels of hamamelose, CA, and CA1P (Andralojc *et al.*, 2002).

CA1P is synthesized from chloroplastic pools of its precursor CA in low light or darkness (Moore *et al.*, 1992; Parry *et al.*, 2008). CA1P has been shown to accumulate at night only in the chloroplast (Moore *et al.*, 1995; Parry *et al.*, 1999), and to bind to Rubisco catalytic sites to inhibit Rubisco activity (Berry *et al.*, 1987; Parry *et al.*, 1997). In *Phaseolus vulgaris*, chloroplastic CA was found to be ~37% of the total CA in illuminated leaves, and after prolonged darkness chloroplast CA levels approached zero, indicating near complete conversion to CA1P (Moore *et al.*, 1992). Interestingly, Moore and colleagues also saw that in several species the pool of CA during light periods greatly exceeded that of CA1P in the dark, indicating either an additional role for extra-chloroplastic CA or very slow turnover of the CA pool. In contrast, in leaves of sugar beet, the opposite was true: CA1P levels in the dark exceeded CA levels in the light. This suggests that beyond the intracellular complexity of CA and CA1P localization, there may be additional species-specific differences in CA metabolism (Moore *et al.*, 1992) and, conceivably, alternative or additional pathways that require or produce CA1P.

**Fig. 2.**
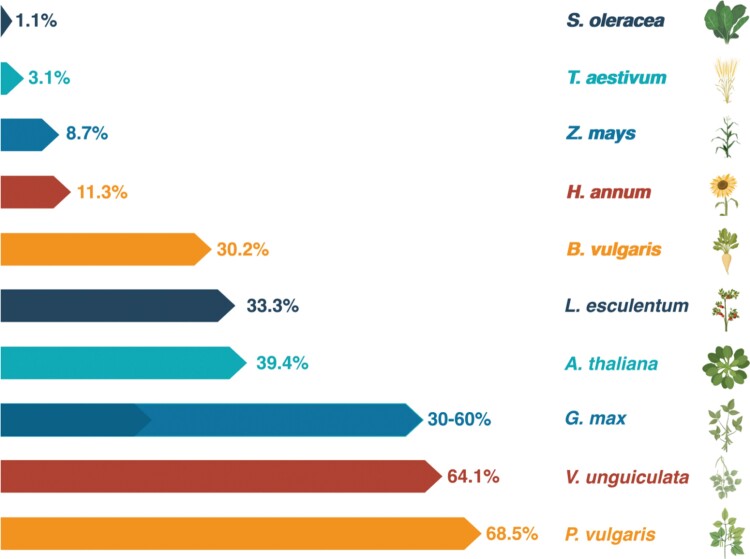
Dark inhibition of Rubisco and CA1P levels vary considerably in different plant species. Dark inhibition values were estimated/calculated from data in Moore *et al.* (1991), with the exception of *Vigna unguiculata* and *Phaseolus vulgaris* (Holbrook *et al.*, 1992), and the range for *Glycine max* cultivar-level differences from Holbrook *et al.* (1994).

Accumulation of CA1P amongst plant species varies greatly, ranging from almost undetectable to >60% dark inhibition of Rubisco in some legumes (Fig. 2). Vu *et al.* (1984) demonstrated that leaves collected from maize and wheat in the dark and high light showed little difference in Rubisco activity. Consistent with this, later CA1P quantification in dark-adapted wheat leaves indicated only enough CA1P to inhibit 7% of Rubisco catalytic sites (Moore *et al.*, 1991); in contrast, leaves of *P. vulgaris* contained sufficient CA1P to potentially inhibit the leaves’ entire Rubisco pool (Charlet *et al.*, 1997). Some C_4_ and Crassulacean acid metabolism (CAM) plants have been shown to contain high levels of the CA1P precursor CA (Moore *et al.*, 1992); indeed, the limited data available suggested strong dark inhibition of Rubisco in CAM plants (Vu *et al.*, 1984), but the C_4_ plants maize, sorghum, and several C_4_*Panicum* species lacked significant dark inhibition (Vu *et al.*, 1984; Moore *et al.*, 1991; Sage and Seemann, 1993). Whilst legumes have the highest levels of dark inhibition reported to date, as highlighted by the major crops used in Fig. 2, there is extraordinary diversity in dark inhibition levels even within the Fabaceae family. An extensive study of 75 species across the Fabaceae (Holbrook *et al.*, 1992), along with detailed work on *Phaseolus* species (Sage, 1993), determined dark inhibition values ranging from 0 to ~70%. These studies also showed the potential for variation within genera, which was further emphasized by follow-on work that showed the potential for intraspecific variation in just six soybean cultivars (Holbrook *et al.*, 1994), whereas accessions of *P. vulgaris* were found to be largely consistent, irrespective of geographical region or cultivation status (Sage, 1993).

## Misfire products of Rubisco’s complex catalytic reaction chemistry

CA1P is the only known sugar phosphate inhibitor actively produced in the cell that regulates Rubisco activity (Andralojc *et al.*, 2012). XuBP, on the other hand, is produced via misprotonation of the enediol intermediate producing the stereoisomer of the substrate RuBP (Kim and Portis, 2004; Pearce, 2006). Rubiscos from diverse lineages including plant, algal, and archaeal sources have been shown to produce XuBP (Zhu and Jensen, 1991; Pearce, 2006). XuBP is produced at a much higher rate than other misfire products (1–3% in high O_2_ and low CO_2_ conditions; Pearce, 2006), and the phosphatase which degrades XuBP has been a subject of study in the recent literature (Bracher *et al.*, 2015, 2017). XuBP is a competitive substrate that functionally acts as an inhibitor due to an exceedingly slow catalytic turnover (*k*_cat_^XuBP^), with Rubisco-catalysed XuBP carboxylation believed to occur at rates fractions of a percent of that of RuBP carboxylation (Yokota, 1991).

In addition to high O_2_/low CO_2_ conditions, XuBP is synthesized by Rubisco at a faster catalytic rate in low pH and higher temperatures (Zhu and Jensen, 1991). XuBP constitutes 74% of all Rubisco misfire products at pH 7.5, whereas only 30% of Rubisco misfire products are XuBP at pH 8.5. Thus, it has been suggested that there may be a greater risk of XuBP inhibition in low light, and the presence of quantifiable XuBP levels *in planta* has only been demonstrated following a brief shift into low light conditions (Zhu and Jensen, 1991).

Two other misfire products (Table 1) are derived from the oxygenase reaction of Rubisco, where H_2_O_2_ elimination from the peroxyketone intermediate generates pentodiulose-1,5-bisphophate (PDBP) and carboxy-tetriol-1,5-bisphosphate (CTBP, a rearrangement of PDBP; Harpel *et al.*, 1995). Non-enzymatic oxidation of RuBP can also produce PDBP and CTBP, and though these inhibitors occur at low frequency (Kim and Portis, 2004; Pearce, 2006), their slow dissociation and tight binding inhibition of catalysis make them an important consideration for inhibition of plant Rubisco in particular (Edmondson *et al.*, 1990; Kane *et al.*, 1998; Pearce, 2006). Pearce and Andrews (2003) found that a catalytically impaired Loop 6 mutant of tobacco Rubisco (Whitney *et al.*, 1999) was also altered in its production of misfire reaction products and its ability to carboxylate XuBP. An increased understanding of Rubisco misfire reactions and the production of inhibitors that need to be ‘cleaned up’ via Rca and sugar phosphatases may yield additional insights if considered in the framework of metabolite repair systems (Linster *et al.*, 2013).

## The sugar phosphatase CA1Pase

The chloroplast contains many phosphatases linked to regulation, and this includes two known sugar phosphatases that degrade Rubisco inhibitors, such as CA1Pase which has been shown in previous studies to be active only in the chloroplast (Gutteridge and Julien, 1989; Moore *et al.*, 1995). Despite its name, CA1Pase has been observed to dephosphorylate other sugar phosphate derivatives, and indeed in some cases has higher affinity (lower *K*_m_ values) for these compared with for CA1P itself (Moore *et al.*, 1995; Andralojc *et al.*, 2002, 2012). Limited data also suggest a correlation between the CA1Pase *K*_m_ for CA1P and CA1P levels. CA1Pase from French bean, a species with high CA1P levels, has a much higher *K*_m_ (430 µM) than CA1Pase from wheat (10 µM), a species with little CA1P (Kingston-Smith *et al.*, 1992; Andralojc *et al.*, 2012). Current knowledge is still limited about CA1Pase specificity and what may be the physiological significance of metabolizing both a synthesized inhibitor (CA1P), and misfire products such as PDBP, particularly as PDBP is similar structurally to RuBP and XuBP, which are not substrates of CA1Pase (Andralojc *et al.*, 2012).

Structurally, CA1Pase is composed of two major domains; the N-terminal domain contains a conserved Arg–His–Gly (RHG) motif identical to the catalytic site of a phosphoglycerate mutase (PGM). This feature is frequently observed for enzymes whose catalytic reaction involves phosphate transfer, including Calvin–Benson–Basham cycle enzymes such as FBPase (Andralojc *et al.*, 2012). Though sharing common sequence features with PGMs, careful examination of the ability of CA1Pases to act on a range of substrates has shown that it lacks true PGM activity, and that a phosphohistidine intermediate is likely to be involved in the reaction mechanism (Andralojc *et al.*, 2012). The C-terminal region of CA1Pase contains a phosphofructokinase (PFK)-like domain, and from studies thus far appears less well conserved than the N-terminal PGM domain, implying more stringent conservation of function in the catalytic site-containing PGM domain (Andralojc *et al.*, 2012).

## The HAD domain sugar phosphatase XuBPase

In the same manner as CA1P, XuBP binds to catalytic sites of Rubisco, inhibiting catalysis. XuBP must first be removed by Rca and then is dephosphorylated by a haloacid dehalogenase-like hydrolase (HAD) domain sugar phosphatase, XuBPase (Bracher *et al.*, 2015). XuBPase was first identified as the product of the *cbbY* gene in the Rubisco operon of *Rhodobacter sphaeroides*, and orthologues of this gene are believed to be universal among photosynthetic organisms, and not present outside this group (Karpowicz *et al.*, 2011; Bracher *et al.*, 2015). The high catalytic efficiency of XuBPase may well be the key reason why measured XuBP concentrations *in planta* are quite low (Zhu and Jensen, 1991). While studies of its properties including regulation and specificity are currently limited, XuBPase has been demonstrated to be highly selective for XuBP over its stereoisomer RuBP (Bracher *et al.*, 2015). XuBP is dephosphorylated to xylulose-5-phosphate which, as well as being non-inhibitory, can be recycled back into the Calvin–Benson–Bassham cycle for RuBP generation (Bracher *et al.*, 2015).

Although they perform a similar function in dephosphorylating a five-carbon sugar phosphate derivative, XuBPase is a HAD domain sugar phosphatase and, thus, evolutionarily unrelated to CA1Pase (Bracher *et al.*, 2015). XuBPase is one of several HAD domain proteins acting to dephosphorylate small molecules in the chloroplast stroma, including 2-phosphoglycolate phosphatase and phosphoserine phosphatase. A closely related HAD domain is also found in the stromal part of the Suppressor of Quenching 1 protein (SOQ1) which is involved in inhibiting a slowly reversible type of non-photochemical quenching (NPQ) (Brooks *et al.*, 2013) that occurs in the light-harvesting complexes associated with PSII (Malnoë *et al.*, 2018). The HAD domain is not necessary for the NPQ function of SOQ1, although it could be involved in its regulation, and the *in vivo* substrate(s) of the SOQ1 HAD domain and its potential impact on Rubisco regulation are currently unknown (Brooks *et al.*, 2013). XuBPase can also act on FBP, though both affinity and catalytic rates with FBP as substrate were dramatically lower than those for XuBP (Bracher *et al.*, 2015).

## Regulation of phosphatases and Rubisco

**Fig. 3.**
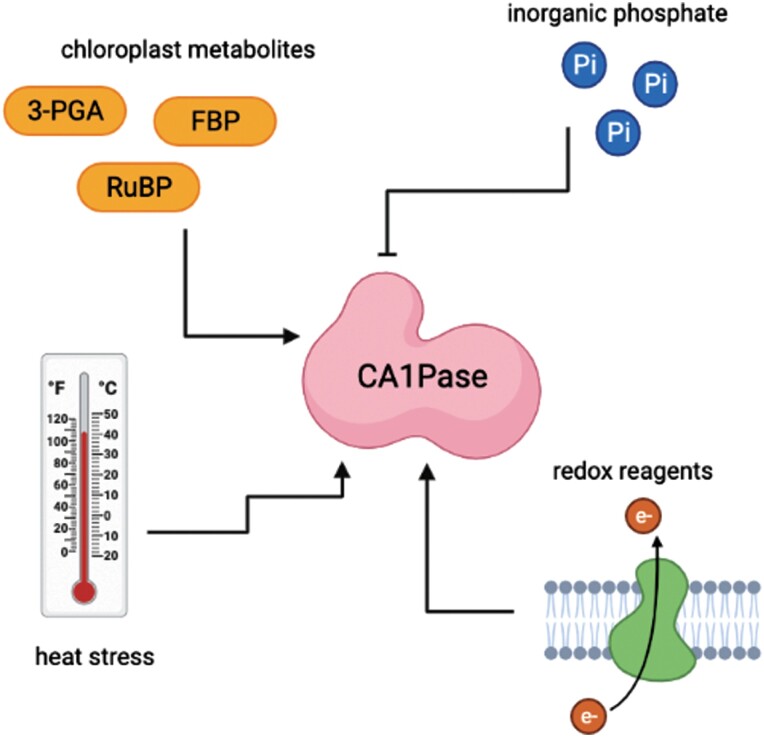
Potential regulators of CA1Pase activity. Summary of potential regulators of CA1Pase activity that have been identified *in vitro.* Many of these observations are consistent with regulation of the light reactions of photosynthesis.

As with other proteins involved in regulating carbon assimilation, such as Rca, sugar phosphatases (particularly CA1Pase) have been shown experimentally to be regulated in multiple ways (Fig. 3). However there do remain unresolved questions around specificity and how conserved these mechanisms may be across species in light of the highly varied levels of their substrates in different plants (see above, Fig. 2). Since CA1Pase is the most well known and studied, its regulation has been explored from several angles to understand its dark–light pattern of activity. Interestingly, but perhaps unsurprisingly, this includes features reminiscent of Rca, which acts in concert with CA1Pase to reactivate Rubisco for maximal activity during the light period.

*In vitro* analyses of CA1Pase activity have shown that several chloroplast metabolites can stimulate activity and increase *V*_max_, including RuBP, FBP, and 3-phosphoglycerate (3-PGA), with as much as a 9-fold increase in the case of FBP (Salvucci and Holbrook, 1989; Holbrook *et al.*, 1991; Andralojc *et al.*, 2012). Curiously, these activators themselves vary in whether they would be expected to increase (e.g. RuBP) or decrease (e.g. FBP) with an increase in light, suggesting that their effect might be concentration dependent. Effective CA1Pase activators consistently contain at least one phosphate group, with either a second phosphate or a carboxyl group in close proximity (Charlet *et al.*, 1997). These metabolites are not substrates of CA1Pase; instead, these phosphorylated effectors are suggested to allosterically interact with the CA1Pase C-terminal PFK-like domain and modulate CA1P dephosphorylation activity (Holbrook *et al.*, 1989; Salvucci and Holbrook, 1991). That these phosphorylated metabolites change during light transitions in the leaf suggests a significant *in vivo* role in regulating CA1Pase activity. Consistent with these observations is the decreased *in vitro* activity of CA1Pase both produced recombinantly and purified from leaves with the addition of inorganic phosphate (Pi); however there is evidence to suggest species differences in this sensitivity (Salvucci and Holbrook, 1989; Holbrook *et al.*, 1991; Charlet *et al.*, 1997; Andralojc *et al.*, 2012). Though the *in vivo* consequences of this are difficult to estimate due to the known variability in leaf Pi content with factors such as leaf age and species (Aziz *et al.*, 2014; Smith *et al.*, 2018), increased CA1Pase activity during illumination is also consistent with light-driven reductions in stromal Pi.

CA1Pase from tobacco has been shown to be resilient to incubation at moderately high temperatures, with activity remaining unaffected after an hour at temperatures up to 30 °C (Holbrook *et al.*, 1991). Above this temperature, post-incubation activity fell precipitously, though CA1Pase thermostability was still higher than that of Rca, another key regulator of Rubisco activity that is known to be thermosensitive. The temperature optimum of CA1Pase activity or expression has to date not received much attention. However, *in vivo* heat stress experiments with wheat, a species which does not possess large amounts of CA1P (Fig. 2), showed a significant increase in CA1Pase activity in leaves after a 5 d heat stress event when the plants had been returned to control conditions (Degen *et al.*, 2021). Redox regulation of chloroplast phosphatases, mediated by thioredoxin, is well established and impacts CA1Pase (Heo and Holbrook, 1999). DTT has been reported as having either a stimulatory or no effect on CA1Pase activity *in vitro* (Holbrook *et al.*, 1991; Heo and Holbrook, 1999; Andralojc *et al.*, 2012) and, during *in vitro* experiments, redox status greatly enhanced protein activity, but this was dependent upon glutathione state, pre-incubation either with other reducing agents such as DTT or air oxidation, and in some cases the assay pH (Heo and Holbrook, 1999; Andralojc *et al.*, 2012).

## Coordination with electron transport

The inhibition of Rubisco by the nocturnal inhibitor CA1P, and subsequent dephosphorylation of CA1P by CA1Pase, has a number of links to the light-dependent side of photosynthesis. Synthesis of CA1P, by an as yet unknown enzyme, occurs in darkness and it progressively inhibits Rubisco in prolonged dark periods when its removal by Rca is limited by stromal ADP/ATP ratios. Increasing light then provides the energy requirements for removal of CA1P by Rca and coincides with promotion of CA1Pase activity to degrade CA1P and render it non-inhibitory. In contrast, treatment with methyl viologen, a PSI electron acceptor, decreased CA1P degradation in the light (Salvucci and Anderson, 1987). In addition, there are well established examples of other light-activated chloroplast phosphatases subject to redox regulation by thioredoxin, and thus linked to electron transport (Heo and Holbrook, 1999). The stimulation of CA1Pase activity by Calvin–Benson–Bassham cycle intermediates also supports coordination between the light reactions, electron transport, and processes which promote the breakdown of the nocturnal inhibitor CA1P.

## A potential role in maintaining Rubisco abundance

Rubisco protein is very abundant in chloroplasts, particularly within C_3_ plants, with plants investing considerable resources to produce Rubisco and the ancillary proteins required for its synthesis and maintaining its activity (reviewed in Carmo-Silva *et al.*, 2015; Bracher *et al.* 2017). Synthesis and assembly of Rubisco have been a rapidly advancing topic in recent years (Hayer-Hartl and Hartl, 2020). There has also been an increased emphasis on the need to better understand the link between enzyme catalytic rates and rates of enzyme protein turnover or replacement (Tivendale *et al.*, 2020; Hanson *et al.*, 2021). This topic is of central importance to Rubisco given the large amounts of protein in C_3_ plants and the central role it plays in carbon metabolism. Rubisco degradation and replacement is an area less understood and might be linked to a protective role for sugar phosphate inhibitors (reviewed in Feller *et al.*, 2008).

**Fig. 4.**
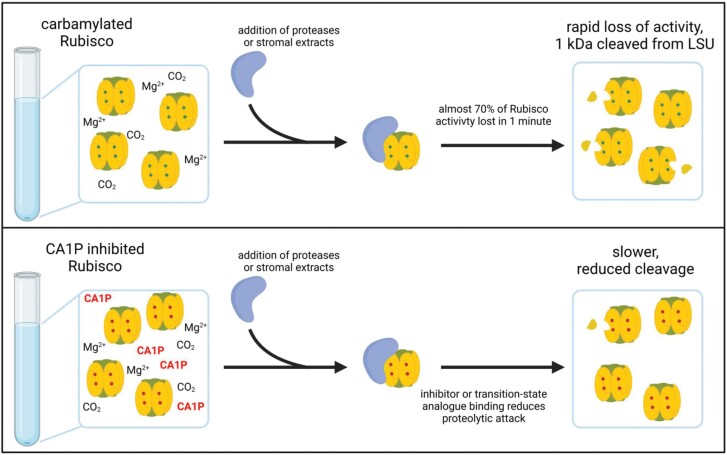
CA1P can limit proteolytic degradation of Rubisco *in vitro*. Illustration of pre-incubation with inhibitors leading to limited ability of proteases to cleave residues off Rubisco large subunits (Khan *et al.*, 1999).

One theory posed for the role of CA1P as a nocturnal inhibitor is to prevent attack of Rubisco by proteases through the conformational changes that occur when the catalytic site changes to bind a sugar phosphate such as CA1P (Fig. 4). Based on *in vitro* experimentation, the closure of Loop 6 has been proposed to limit the accessibility of the large subunit for proteolysis, which would conserve Rubisco protein (Khan *et al.*, 1999). The same authors suggested that, upon illumination or alleviation of stress, the inhibitor would be removed from the catalytic site and Rubisco would be readily available for catalysis. In that study, CA1P did not specifically inhibit the protease, and pre-incubation with CA1P greatly slowed proteolysis of the large subunit by trypsin or carboxypeptidase A, especially in the presence of Mg^2+^ and CO_2_, to form a carbamate within the catalytic site prior to CA1P binding. Stromal protease extracts were also unable to degrade Rubisco that had been activated and incubated with CA1P (Khan *et al.*, 1999). The authors theorized that during the day high levels of carbamylation combined with RuBP and the binding of daytime inhibitors such as misfire products could confer protection from proteolysis, a role which at night when RuBP is low would be taken over by CA1P. Supporting this idea is the ability of CA1P to limit degradation in other Rubiscos, and work with either CABP or RuBP that saw reduced cleavage by proteases through pre-incubation with sugar phosphates (Houtz and Mulligan, 1991; Chen and Spreitzer, 1992). Tobacco plants deficient in Rca, which allowed accumulation of inhibition by tight binding inhibitors, were also found to accumulate high levels of Rubisco that was less active (He *et al.*, 1997).

As Khan *et al.* (1999) noted, in many species there is insufficient CA1P to bind all Rubisco catalytic sites. They did, however, see variability in how some proteases attacked Rubisco from different species. If susceptibility to proteolysis is species specific this may in part explain the large differences in CA1P content amongst plants. Curiously, this work saw wheat Rubisco protected by binding of CA1P, despite wheat being a species which shows comparatively little dark inhibition and CA1P content (Fig. 2). In addition, surprising results with CA1Pase in wheat were observed with plants overexpressing CA1Pase (Lobo *et al.*, 2019). Overexpression of CA1Pase was hypothesized to lower inhibitor content and lead to increased activation status of Rubisco. This was found to be true; however, unexpectedly, Rubisco abundance in these plants decreased by as much as 60%, leading to reductions in growth and yield (Lobo *et al.*, 2019). The trade-off between Rubisco abundance and activation status has been observed in many species including wheat, where increases in activation status are negatively correlated with Rubisco abundance (Carmo-Silva *et al.*, 2017), and in transgenic rice overexpressing Rca (Fukayama *et al.*, 2012). Combined with observations discussed above, this result adds weight to theories around protection from degradation. However, many questions remain, particularly around the possibility that CA1Pase activity may be linked to Rubisco synthesis/degradation (Feller *et al.*, 2008), and whether the results observed *in vitro* are representative of the interaction between stromal proteases and inhibited Rubisco.

## Conclusion

The inhibition of Rubisco by tightly binding sugar phosphates, either actively synthesized or derived from misfire of its complex reaction mechanism, can have large impacts on Rubisco activity by limiting carboxylation capacity. Key to this regulation of Rubisco activity is the action of Rca in removing these inhibitors from the Rubisco catalytic site, followed by their dephosphorylation by sugar phosphatases. Despite extensive study of dark inhibition of Rubisco by CA1P, many questions remain about the role of this seemingly ubiquitous, yet highly variable, process. The potential for a role in modulating Rubisco abundance as well as activity may make this a necessary consideration for manipulating Rubisco *in planta* for improved photosynthesis. Regulation of the phosphatases CA1Pase and XuBPase responsible for inhibitor degradation also warrants deeper investigation of these highly conserved components of Rubisco regulation. This conservation, the rapid development of CRISPR/Cas9 technologies in plants, and the large variation evident inter- and intraspecies provide encouragement for better understanding this regulation as well as its potential role in improving photosynthetic efficiency and crop productivity.
